# Use of the head-twitch response to investigate the structure–activity relationships of 4-thio-substituted 2,5-dimethoxyphenylalkylamines

**DOI:** 10.1007/s00213-022-06279-2

**Published:** 2022-12-07

**Authors:** Adam L. Halberstadt, Dino Luethi, Marius C. Hoener, Daniel Trachsel, Simon D. Brandt, Matthias E. Liechti

**Affiliations:** 1 Department of Psychiatry, University of California San Diego, 9500 Gilman Dr, La Jolla, CA 92093-0804 USA; 2 Research Service, VA San Diego Healthcare System, San Diego, CA USA; 3 Division of Clinical Pharmacology and Toxicology, Department of Biomedicine and Department of Pharmaceutical Sciences, University Hospital Basel and University of Basel, Basel, Switzerland; 4 pRED, Roche Innovation Center Basel, Neuroscience Research, F. Hoffmann-La Roche Ltd, Basel, Switzerland; 5 ReseaChem GmbH, Burgdorf, Switzerland; 6 School of Pharmacy and Biomolecular Sciences, Liverpool John Moores University, Liverpool, UK

**Keywords:** Psychedelic, Hallucinogen, 5-HT_2A_ receptor, Head-twitch response, Phenethylamine, Mice

## Abstract

**Rationale:**

4-Thio-substituted phenylalkylamines such as 2,5-dimethoxy-4-ethylthiophenethylamine (2C-T-2) and 2,5-dimethoxy-4-*n*-propylthiophenethylamine (2C-T-7) produce psychedelic effects in humans and have been distributed as recreational drugs.

**Objectives:**

The present studies were conducted to examine the structure–activity relationships (SAR) of a series of 4-thio-substituted phenylalkylamines using the head twitch response (HTR), a 5-HT_2A_ receptor-mediated behavior induced by psychedelic drugs in mice. The HTR is commonly used as a behavioral proxy in rodents for human psychedelic effects and can be used to discriminate hallucinogenic and non-hallucinogenic 5-HT_2A_ agonists.

**Methods:**

HTR dose–response studies with twelve different 4-thio-substituted phenylalkylamines were conducted in male C57BL/6 J mice. To detect the HTR, head movement was recorded electronically using a magnetometer coil and then head twitches were identified in the recordings using a validated method based on artificial intelligence.

**Results:**

2C-T, the parent compound of this series, had relatively low potency in the HTR paradigm, but adding an α-methyl group increased potency fivefold. Potency was also increased when the 4-methylthio group was extended by one to three methylene units. Fluorination of the 4-position alkylthio chain, however, was detrimental for activity, as was the presence of a 4-allylthio substituent versus a propylthio group. 2C-T analogs containing a 4-benzylthio group showed little or no effect in the HTR paradigm, which is consistent with evidence that bulky 4-substituents can dampen agonist efficacy at the 5-HT_2A_ receptor. Binding and functional studies confirmed that the compounds have nanomolar affinity for 5-HT_2_ receptor subtypes and act as partial agonists at 5-HT_2A_.

**Conclusions:**

In general, there were close parallels between the HTR data and the known SAR governing activity of phenylalkylamines at the 5-HT_2A_ receptor. These findings further support the classification of 2C-T compounds as psychedelic drugs.

**Supplementary Information:**

The online version contains supplementary material available at 10.1007/s00213-022-06279-2.

## Introduction

In recent years, there has been increasing scientific and medical interest in psychedelic drugs such as psilocybin, lysergic acid diethylamide (LSD), and mescaline. The focus on these agents has been driven, in large part, by recognition that they may possess therapeutic utility for multiple psychiatric indications (Bogenschutz and Ross 2018). For example, promising results have been reported in clinical trials evaluating psilocybin for depression (Carhart-Harris et al. 2021; Davis et al. 2021), existential distress (Griffiths et al. 2016; Ross et al. 2016), nicotine dependence (Johnson et al. 2014, 2017), and alcoholism (Bogenschutz et al. 2015). The 5-HT_2A_ receptor is believed to be the primary target for psychedelic drugs in the brain (Halberstadt 2015; Nichols 2016). The intensity of the psychedelic state induced by psilocybin is correlated with 5-HT_2A_ occupation in the CNS, measured using the PET radioligand [^11^C]Cimbi-36 (Madsen et al. 2019). In addition, 5-HT_2A_ antagonists block the characteristic hallucinogenic effects induced by psilocybin and LSD (Holze et al. 2021; Kometer et al. 2013; Preller et al. 2017; Vollenweider et al. 1998).

Most psychedelic drugs are derived from the tryptamine and phenylalkylamine structural scaffolds. The structure–activity relationships (SAR) of psychedelic drugs with a phenylalkylamine structure have received considerable attention over the last five decades. Although mescaline and other phenylalkylamines with a 3,4,5-substitution pattern are active in human as psychedelics (Beringer 1927; Peretz et al. 1955), chemists have focused on the 2,4,5-pattern because it tends to maximize human potency. For example, 3,4,5-trimethoxyamphetamine (TMA) is active at a dose range of 100–250 mg, whereas its 2,4,5-regioisomer (2,4,5-trimethoxyamphetamine, TMA-2) is active at 20–40 mg (Shulgin and Shulgin 1991). Replacement of the 4-methoxy group in TMA-2 with a 4-methylthio substituent increases potency even further; 2,5-dimethoxy-4-methylthioamphetamine (ALEPH) is active at 5–15 mg (Shulgin and Nichols 1978; Shulgin and Shulgin 1991). The ability of a 4-position sulfur atom to increase potency is consistent with evidence that phenylalkylamines containing a 2,5-dimethoxy-substitution pattern and a lipophilic 4-substituent have especially high potency (Nichols 2018).

**Fig. 1:**
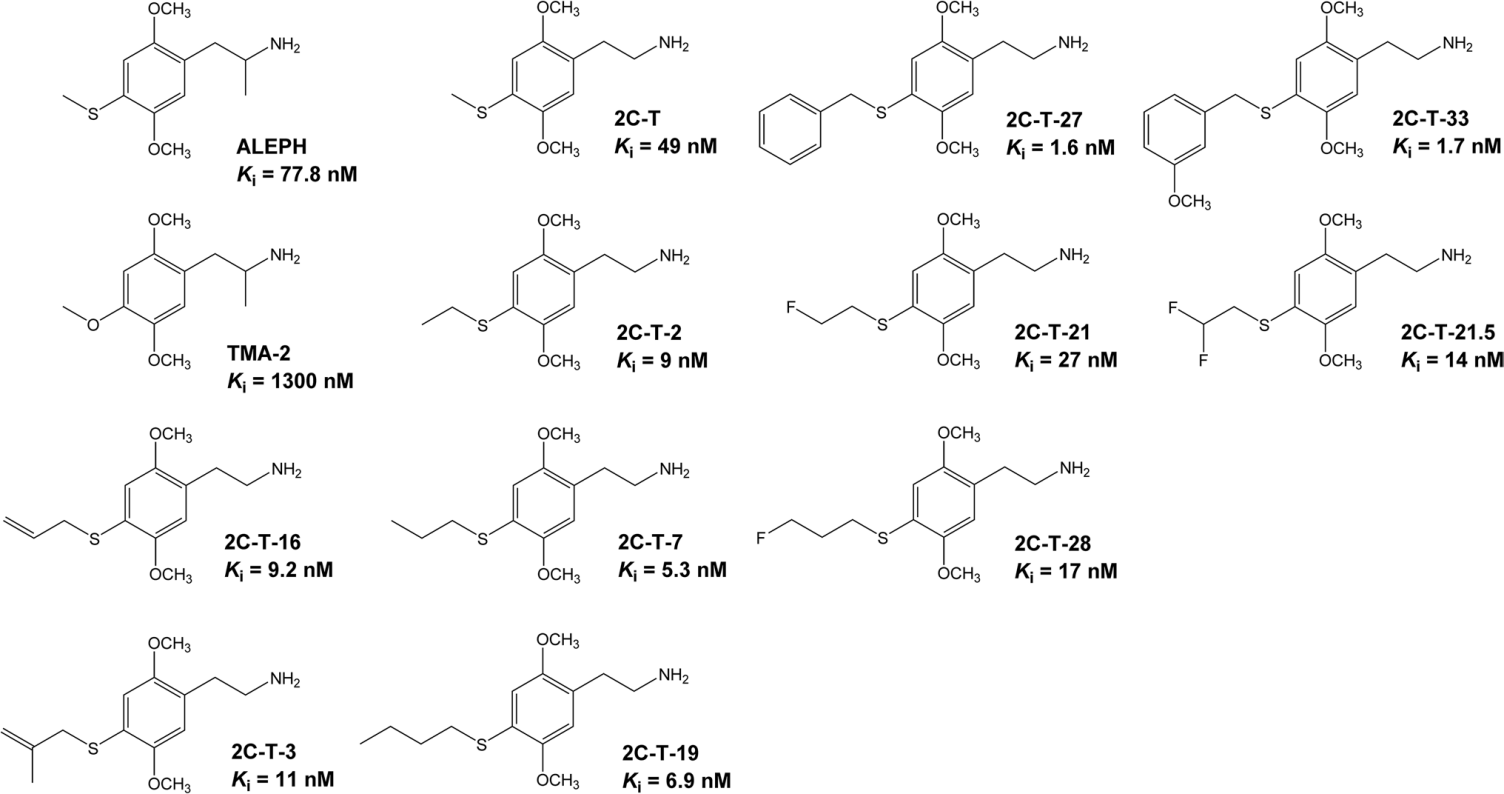
Chemical structures of 2,5-dimethoxy-4-methylthiophenethylamine (2C-T) and other sulfur-substituted phenylalkylamines. Published binding affinities for the human 5-HT_2A_ receptor labeled with [^3^H]ketanserin are also included (the binding data were taken from: Kolaczynska et al. (2019); Luethi et al. (2018); Rickli et al. (2015)). The binding data for ALEPH and 2C-T-21 are from the present investigation

In addition to ALEPH, several other phenylalkylamines containing a 4-position sulfur atom have been evaluated in humans. The compounds are derivatives of 2,5-dimethoxyphenethylamine (2C-H). 2,5-Dimethoxy-4-methylthiophenethylamine (2C-T), the α-desmethyl homologue of ALEPH, is active at 60–100 mg (Shulgin and Shulgin 1991; Shulgin et al. 1991). Lengthening the 4-methylthio group in 2C-T can increase potency to a considerable degree. 2C-T-2 and 2C-T-7, the 4-ethylthio and 4-*n*-propylthio homologues, are active at 12–25 mg and 10–30 mg, respectively (Shulgin and Shulgin 1991). 2C-T-2 and 2C-T-7 have been distributed in the USA and in European countries as recreational drugs (Curtis et al. 2003; de Boer and Bosman 2004; Schifano et al. 2005; King et al. 2014). As shown in Fig. 1, phenylalkylamines containing 4-fluoroalkylthio and 4-benzylthio substituents have also been investigated (Luethi et al. 2018; Shulgin and Shulgin 1991; Trachsel et al. 2013), but relatively little is known about their activity in humans and in other species.

Although several different animal models can be used to evaluate whether compounds produce psychedelic-like behavioral effects, few studies have been conducted with sulfur-containing phenylalkylamines. Over the last few decades, drug discrimination has been one of the primary models used to investigate the behavioral effects produced by psychedelic drugs in laboratory animals (Glennon et al. 1983). In drug discrimination studies, animals are trained to press one of two levers depending on whether they receive vehicle control or a training drug, often LSD or 2,5-dimethoxy-4-methylamphetamine (DOM). 2C-T-7 produces hallucinogen-like effects in rats and monkeys trained to discriminate DOM (Khorana et al. 2004; Li et al. 2010, 2008). When tested in rats trained to discriminate LSD or DMT, however, 2C-T-7 and 2C-T-2 produced partial generalization but did not fully substitute for the training drug (Eshleman et al. 2014; Fantegrossi et al. 2005). Rats can also be trained to discriminate 2C-T-7 from saline using two-lever drug discrimination procedures and substitution is blocked by the selective 5-HT_2A_ antagonist M100907, indicating the stimulus cue is mediated by the 5-HT_2A_ receptor (Fantegrossi et al. 2005).

2C-T-7 has also been shown to induce the head-twitch response (HTR) in mice (Fantegrossi et al. 2005). The HTR is a rapid rotational head shaking induced by psychedelic drugs via activation of the 5-HT_2A_ receptor (Canal and Morgan 2012). Studies commonly use the HTR as a behavioral proxy in mice for human hallucinogen effects because it can reliably distinguish hallucinogenic and non-hallucinogenic 5-HT_2A_ receptor agonists (Gonzalez-Maeso et al. 2007). For example, LSD induces the HTR, whereas its non-hallucinogenic analogue lisuride does not induce the response (Gonzalez-Maeso et al. 2007; Halberstadt and Geyer 2013). HTR potencies in male C57BL/6 J mice are strongly correlated with hallucinogenic potencies in humans and ED_50_ values from drug discrimination studies using either DOM or LSD as the training drug (Halberstadt et al. 2020).

Given the lack of information about the behavioral activity of 2C-T and other 2,5-dimethoxyphenylalkylamines containing a sulfur atom in the 4-position, HTR experiments were conducted in male C57BL/6 J mice to assess their interactions with the 5-HT_2A_ receptor in vivo. The HTR experiments were designed to address two questions. First, does each molecule have an LSD-like behavioral profile? Second, how does the identity of the 4-substituent influence activity in the HTR paradigm? Although the HTR has traditionally been assessed by direct observation or video recording, we recently developed and validated an electronic assessment technique that can detect head twitches with high sensitivity and specificity. In this approach, activity is recorded using a head-mounted magnet and a magnetometer coil (Halberstadt and Geyer 2013), and then head twitches are identified in the recordings using artificial intelligence (Halberstadt 2020). These procedures were used to evaluate the effects of 2C-T and ten analogs on the HTR (the chemical structures of the molecules are illustrated in Fig. 1). Most of the analogs have been shown to bind to 5-HT_2A_ receptors with nanomolar affinity (Fantegrossi et al. 2005; Luethi et al. 2018; Rickli et al. 2015). However, two of the compounds were not evaluated previously, so binding and functional assays were performed to assess their interactions with 5-HT_2_ receptor subtypes.

## Materials and methods

### Drugs

2,5-Dimethoxy-4-methylthiophenethylamine hydrochloride (2C-T), 2,5-dimethoxy-4-ethylthiophenethylamine hydrochloride (2C-T-2), 2,5-dimethoxy-4-methallylthiophenethylamine hydrochloride (2C-T-3), 2,5-dimethoxy-4-*n*-propylthiophenethylamine hydrochloride (2C-T-7), 4-allylthio-2,5-dimethoxyphenethylamine hydrochloride (2C-T-16), 4-*n*-butylthio-2,5-dimethoxyphenethylamine hydrochloride (2C-T-19), 2,5-dimethoxy-4-(2-fluoroethylthio)phenethylamine hydrochloride (2C-T-21), 4-(2,2-difluoroethylthio)-2,5-dimethoxyphenethylamine hydrochloride (2C-T-21.5), 4-benzylthio-2,5-dimethoxyphenethylamine hydrochloride (2C-T-27), 4-(3-fluoropropylthio)-2,5-dimethoxyphenethylamine hydrochloride (2C-T-28), and 2,5-dimethoxy-4-(3-methoxybenzylthio)phenethylamine hydrochloride (2C-T-33) were provided by ReseaChem GmbH (Burgdorf, Switzerland). Purity was > 98.5% by high-performance liquid chromatography (HPLC). 2,5-Dimethoxy-4-methylthioamphetamine hydrochloride (ALEPH) was available from previous studies.

### Animals

Male C57BL/6 J mice (Jackson Laboratory, Bar Harbor, ME) were housed in a vivarium at the University of California San Diego (UCSD), which is an AAALAC-approved animal facility that complies with Federal and State requirements for care and treatment of laboratory animals. The mice (6–8 weeks old) were housed in a climate-controlled room with a reversed light-cycle (lights on at 19:00 h, off at 07:00 h) up to 4 animals per cage. Food and water were provided ad libitum, except during behavioral testing which occurred between 10:00 and 18:00 h. All experiments were conducted according to NIH guidelines and were approved by the UCSD animal care committee.

### Head-twitch response studies

Head movement was recorded using a head-mounted magnet and a magnetometer coil (Halberstadt and Geyer 2013). Briefly, mice were anesthetized, a small incision was made in the scalp, and a neodymium magnet was attached to the dorsal surface of the cranium using dental cement. Following a 1–2-week recovery period, behavioral experiments were conducted in a well-lit room with at least 7 days between sessions to avoid any carryover effects. Mice were treated with vehicle or test compound and then placed in a glass cylinder surrounded by a magnetometer coil and activity was recorded for 30 min. All test substances were administered IP dissolved in saline using an injection volume of 5 mL/kg. Coil voltage was filtered (5–10 kHz lowpass), digitized (20 kHz sampling rate), and saved to disk using a Powerlab/8SP with LabChart v 7.3.2 (ADInstruments, Colorado Springs, CO). To detect head twitches, events in the recordings were converted into scalograms using a wavelet transform and then the images were classified using a multistage approach combining the deep convolutional neural network (CNN) *ResNet-50* with a support vector machine (SVM) algorithm (Halberstadt 2020). HTR counts were analyzed using a one-way Welch analysis of variance (ANOVA). Post hoc pairwise comparisons were performed using Dunnett’s test. Significance was demonstrated using an α-level of 0.05. Median effective doses (ED_50_ values) and 95% confidence intervals were calculated using nonlinear regression (Prism ver. 9.0.2, GraphPad Software Inc, San Diego, CA, USA).

### 5-HT receptor binding

5-HT receptor binding affinities were assessed as previously described in detail (Luethi et al. 2018). In brief, membrane preparations of transiently transfected HEK 293 cells were incubated with selective radioligands at concentrations equal to the corresponding *K*_d_ and then ligand displacement by the test drugs was measured. The difference between the total binding and the nonspecific binding determined in the presence of selective competitors was defined as specific binding. The following radioligands and competitors, respectively, were used: 0.40 nM [^3^H]ketanserin and 10 μM spiperone (serotonin 5-HT_2A_ receptor), 1.4 nM [^3^H]mesulergine and 10 μM mianserin (serotonin 5-HT_2C_ receptor). The IC_50_ values of the radioligand binding assays were determined using nonlinear regression curves for every drug in a one-site model. *K*_i_ values corresponding to the dissociation constants were calculated with the Cheng-Prusoff equation.

### Agonist activity at the 5-HT_2A_ receptor

NIH-3T3 cells stably expressing the human 5-HT_2A_ receptor were incubated in HEPES-Hank’s Balanced Salt Solution (HBSS) buffer (Gibco, Zug, Switzerland; 70,000 cells/100 μL) for 1 h at 37 °C in poly-D-lysine-coated 96-well plates. Thereafter, 100 μL of dye solution (fluorescence imaging plate reader [FLIPR] calcium 5 assay kit dye solution; Molecular Devices, Sunnyvale, CA, USA) was added to each well and the plates were incubated for 1 h at 37 °C. The plates were then placed into a FLIPR and 25 μL of the test drugs diluted in HEPES-HBSS containing 250 mM probenicid was added to each well online. The increase in fluorescence was measured for 51 s, and EC_50_ values were derived from the concentration–response curves using nonlinear regression.

### Agonist activity at the 5-HT_2B_ receptor

HEK 293 cells stably expressing the human 5-HT_2B_ receptor were incubated overnight in poly-D-lysine-coated 96-well plates at a density of 50,000 cells per well and 37 °C. Thereafter, the growth medium was removed by snap inversion and 200 μL of no wash dye (FLIPR calcium 6 assay kit; Molecular Devices, Sunnyvale, CA, USA) was added to each well. The plates were incubated for 2 h at 37 °C and then placed into a FLIPR. Fifty microliters of the test drugs diluted in assay buffer was added to each well online and the increase in fluorescence was measured for 51 s. EC_50_ values were derived from the concentration–response curves using nonlinear regression.

## Results

### Automated detection of the head-twitch response in mice

Although the HTR is usually detected in mice and rats by direct observation (Corne and Pickering 1967; Corne et al. 1963; Silva and Calil 1975), a semi-automated method has been developed to assess the behavior using a head-mounted magnet and a magnetometer coil (Halberstadt and Geyer 2013). The magnetometer-based approach has proven very effective for SAR studies with psychedelic drugs in mice (Halberstadt et al. 2019a, 2019b; Klein et al. 2021, 2018; Marcher-Rorsted et al. 2020; Nichols et al. 2015). More recently, procedures were developed to automate the detection of head twitches using scalograms and deep learning (Halberstadt, 2020). The deep learning technique was used to analyze the HTR experiments performed with the sulfur-substituted phenylalkylamines. To further validate these automated HTR detection procedures, head twitches were also identified by manual analysis of the recordings using published methods (Halberstadt and Geyer 2013; 2014). The results are summarized in Table 1. In the published validation experiments, the multistaged CNN-SVM approach identified 99.4% of the HTR induced by various psychedelic drugs (Halberstadt, 2020). Performance in the present studies was very similar, with 99.3% of the HTR detected (10,411 out of 10,481 total head twitches) across the twelve experiments (*R* = 1, *p* < 0.0001).

**Table 1:** Summary of the performance of the automated HTR detection procedures

Compound	*N*	Manual HTR Count	Automated detection	
			Number of HTR detected	Percent detected
ALEPH	30	1,464	1,455	99.4%
2C-T	35	1,718	1,707	99.4%
2C-T-2	28	479	470	98.1%
2C-T-3	30	976	966	99.0%
2C-T-7	27	930	922	99.1%
2C-T-16	25	1,549	1,538	99.3%
2C-T-19	25	420	420	100%
2C-T-21	28	931	925	99.4%
2C-T-21.5	25	1,002	999	99.7%
2C-T-27	30	237	236	99.6%
2C-T-28	27	592	590	99.7%
2C-T-33	25	183	183	100%
Total:	335	10,481	10,411	99.3%

### Effect of 4-thio-substituted 2,5-dimethoxyphenylalkylamines in mice

**Fig. 2:**
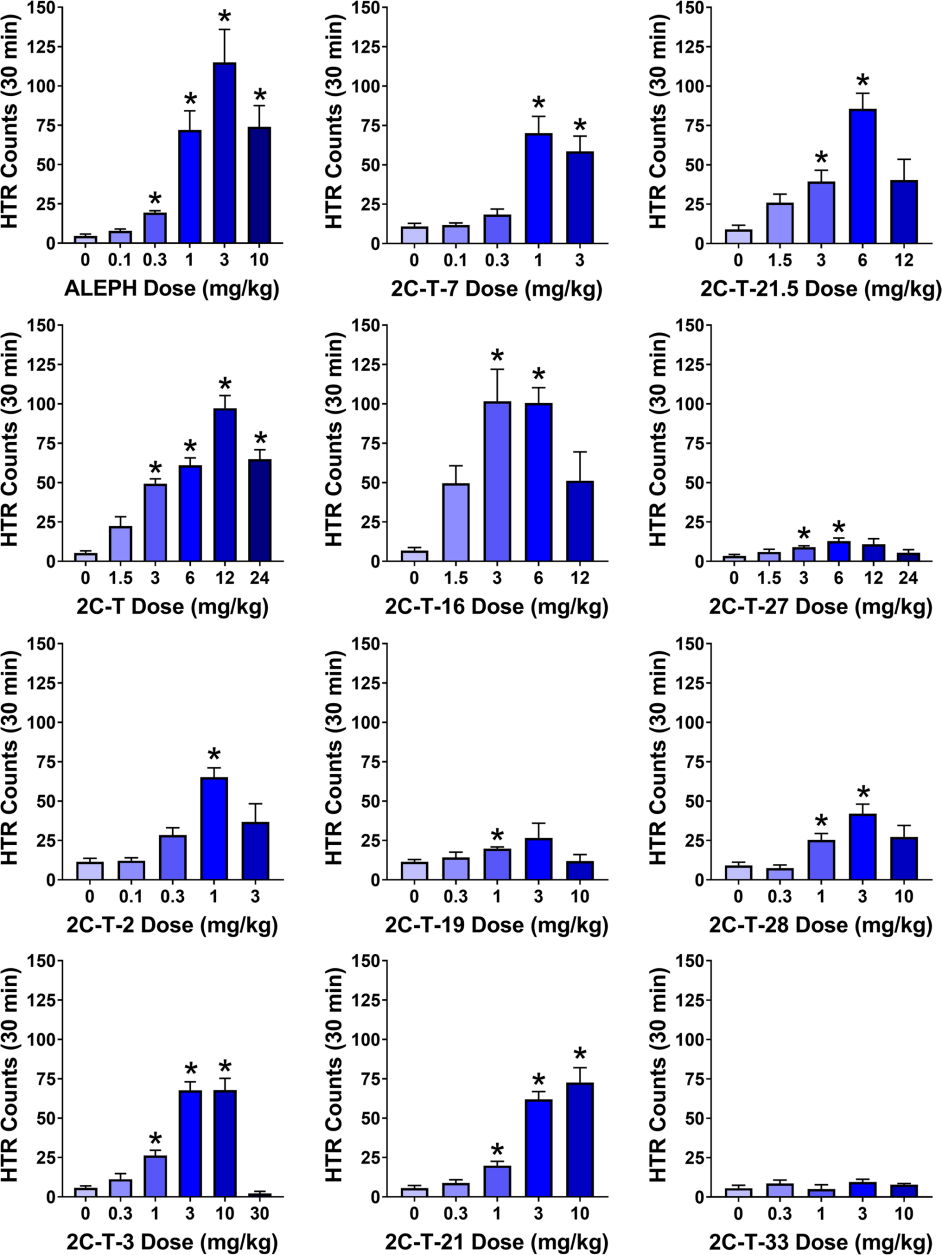
Effect of sulfur-substituted phenylalkylamines on the head-twitch response (HTR). Each compound was injected IP and then activity was recorded continuously for 30 min. Data are presented as group means ± SEM for the entire test session. *p < 0.05, significant difference compared to the vehicle control group (Dunnett’s T3 multiple comparisons test)

As shown in Fig. 2, the 4-thio-substituted derivatives of 2C-H and 2,5-dimethoxyamphetamine (2,5-DMA) induce head twitches in C57BL/6 J mice. Similar to other psychedelic drugs (e.g., Fantegrossi et al. 2010; Halberstadt et al. 2020), the compounds had biphasic, bell-shaped dose–response functions in the HTR experiments. Experimental details, median effective doses (ED_50_ values), and statistical results for each compound are summarized in Table 2 and Table S1. ALEPH induced the HTR in mice with an ED_50_ = 0.80 mg/kg, which is equivalent to 2.88 µmol/kg. In comparison, when tested in a previous experiment using similar methods, the 4-methoxy analog TMA-2 induced the HTR with an ED_50_ of 12.4 µmol/kg (Halberstadt et al. 2019a). Replacing the 4-methoxy group in TMA-2 with a 4-methylthio group increased potency fourfold. There is reportedly a similar difference in potency between TMA-2 (20–40 mg p.o.) and ALEPH (5–10 mg p.o.) in humans (Shulgin and Shulgin 1991).

**Table 2:** Potency of phenylalkylamines in HTR experiments conducted in C57BL/6 J mice. Binding affinities for human 5-HT_2A_ sites labeled with [^3^H]ketanserin, as well as the dose range reported to produce psychedelic effects in humans, are also included for reference purposes

Compound	ANOVA	ED_50_ mg/kg (95% CI)	ED_50_ µmol/kg (95% CI)	5-HT_2A_ *K*_i_ (nM)	Human potency range
TMA-2		2.79 (1.94–4.01) ^[1]^	12.4 (8.6–17.8) ^[1]^	1300 ^[2]^	20–30 mg ^[5]^
ALEPH	*W*(5,10.78) = 24.83, *p* < 0.0001	0.80 (0.53–1.20)	2.88 (1.91–4.32)	77.8	5–15 mg ^[5]^
2C-T	*W*(5,11.72) = 70.03, *p* < 0.0001	3.65 (3.09–4.32)	13.8 (11.7–16.4)	49 ^[3]^	60–100 mg ^[5]^
2C-T-2	*W*(4,10.02) = 18.13, *p* < 0.0001	0.40 (0.28–0.57)	1.44 (1.01–2.05)	9 ^[4]^	12–25 mg ^[5]^
2C-T-3	*W*(5,9.97) = 37.16, *p* < 0.0001	1.86 (1.45–2.39)	6.12 (4.77–7.87)	11 ^[3]^	12–25 mg ^[6]^
2C-T-7	*W*(4,10.12) = 11.69, *p* = 0.0008	0.62 (0.38–0.99)	2.12 (1.30–3.39)	5.3 ^[3]^	15–40 mg ^[5]^
2C-T-16	*W*(4,8.33) = 25.78, *p* < 0.0001	1.68 (1.17–2.43)	5.80 (4.04–8.38)	9.2 ^[3]^	10–25 mg ^[6]^
2C-T-19	*W*(4,9.27) = 4.80, *p* = 0.0228	1.00 (0.48–2.11)	3.27 (1.57–6.90)	6.9 ^[3]^	
2C-T-21	*W*(4,10.75) = 35.21, *p* < 0.0001	1.65 (1.19–2.27)	5.58 (4.02–7.67)	27	8–12 mg ^[5]^
2C-T-21.5	*W*(4,9.16) = 14.98, *p* = 0.0005	3.13 (2.48–3.93)	9.97 (7.90–12.5)	14 ^[3]^	12–30 mg ^[6]^
2C-T-27	*W*(5,10.87) = 5.05, *p* = 0.0122	2.48 (1.46–4.22)	7.30 (4.30–12.4)	1.6 ^[3]^	80–130 mg ^[6]^
2C-T-28	*W*(4,10.12) = 9.73, *p* = 0.0017	1.08 (0.62–1.87)	3.49 (2.00–6.04)	17 ^[3]^	8–30 mg ^[6]^
2C-T-33	*W*(4,9.35) = 0.74, *p* = 0.5897	*ND*	*ND*	1.7 ^[3]^	

[1] Halberstadt et al. (2019a); [2] Kolaczynska et al. (2019), [3] Luethi et al. (2018); [4] Rickli et al. (2015); [5] Shulgin and Shulgin (1991); [6] Trachsel et al. (2013)

*ND*, not determined

2C-T, the α-desmethyl homologue of ALEPH, acts as a psychedelic drug in humans (Shulgin and Shulgin 1991). As anticipated, 2C-T induces the HTR in mice (ED_50_ = 13.8 µmol/kg), but has fivefold lower potency than ALEPH. Note that in humans, 2C-T has about tenfold lower potency than ALEPH as a psychedelic drug. These results are consistent with published HTR data showing that the addition of an α-methyl group increases the potency of 2,4,5-trisubstituted phenylethylamines by approximately 3–5-fold (Halberstadt et al. 2019b).

Increasing the length of the 4-alkylthio chain in 2C-T produced a marked increase in potency. Activity peaked with 2C-T-2 (ED_50_ = 1.44 µmol/kg), the two-carbon homologue, which has ten times the potency of 2C-T. 2C-T-7 (ED_50_ = 2.12 µmol/kg), the three-carbon homologue, has seven times the potency of 2C-T, whereas the four-carbon homologue 2C-T-19 (ED_50_ = 3.27 µmol/kg) has four times the potency of 2C-T. Interestingly, the maximum number of head twitches induced by 2C-T-19 during the 30-min assessment period (mean ± SEM = 26.6 ± 9.4) was relatively low compared to homologues with shorter 4-alkylthio chains (2C-T = 97.2 ± 8.1; 2C-T-2 = 65.2 ± 5.9; 2C-T-7 = 70.2 ± 10.6), indicating that relatively long 4-position alkylthio chains have detrimental effects on HTR intensity. Replacing the 4-*n*-propylthio group in 2C-T-7 with a 4-allylthio group (2C-T-16, ED_50_ = 5.80 µmol/kg) or a 4-(β-methylallyl)thio group (2C-T-3, ED_50_ = 6.12 µmol/kg) is also detrimental for activity in the HTR paradigm, reducing potency by more than 50%.

Several fluorinated analogues were evaluated. Monofluorination of the 4-ethylthio group in 2C-T-2 results in a fourfold drop in potency (2C-T-21, ED_50_ = 5.58 µmol/kg). Difluorination, by contrast, reduces the potency of 2C-T-2 by almost an order of magnitude (2C-T-21.5, ED_50_ = 9.97 µmol/kg). Monofluorination of the 4-*n*-propylthio group in 2C-T-7 is also detrimental for activity, but the reduction in potency was less than twofold (2C-T-28, ED_50_ = 3.49 µmol/kg).

Experiments were also conducted with 2C-T-27 and 2C-T-33, which contain 4-benzylthio and 4-(3-methoxy)benzylthio groups, respectively. These 4-benzylthio substituted compounds produced weak responses in the HTR paradigm (see Fig. 2). At doses ranging from 0.3–10 mg/kg, 2C-T-33 did not produce a significant increase in HTR counts over baseline levels (*W*(4,9.35) = 0.74, *p* = 0.5897). Although 2C-T-27 was active (*W*(5,10.87) = 5.05, *p* = 0.0122) and increased HTR counts with ED_50_ = 7.30 µmol/kg, the magnitude of the response was relatively weak in comparison to most of the compounds that were tested from this series.

### Interactions with 5-HT_2A_, 5-HT_2B_, and 5-HT_2C_ receptors

With the exception of ALEPH-1 and 2C-T-21, the phenylalkylamines evaluated above were previously found to bind to and activate 5-HT_2_ receptor subtypes (Fantegrossi et al., 2005; Eshleman et al., 2014; Rickli et al., 2015; Luethi et al., 2018). Binding and functional assays were therefore performed with ALEPH-1 and 2C-T-21 to assess their interaction with human 5-HT_2A_, 5-HT_2B_, and 5-HT_2C_ receptors. ALEPH-1 binds to h5-HT_2A_ and h5-HT_2C_ receptors with *K*_i_ values of 77.8 ± 18.8 nM and 355 ± 76 nM, respectively, and acts as a partial agonist at h5-HT_2A_ (EC_50_ = 10.3 ± 0.6 nM, *E*_max_ = 80.0 ± 2.3% relative to 5-HT) and h5-HT_2B_ (EC_50_ = 19.2 ± 1.7 nM, *E*_max_ = 51.0 ± 2.6%) in calcium flux assays. 2C-T-21 binds to h5-HT_2A_ and h5-HT_2C_ receptors with *K*_i_ values of 27.7 ± 6.2 nM and 72.7 ± 10.6 nM, respectively, and acts as a partial agonist at h5-HT_2A_ (EC_50_ = 9.7 ± 0.1 nM, *E*_max_ = 69.0 ± 4.8%) and h5-HT_2B_ (EC_50_ = 102 ± 24 nM, *E*_max_ = 32.0 ± 1.8%).

## Discussion

The present investigation was conducted to evaluate the behavioral response to 2C-T and several other 4-thio-substituted 2,5-dimethoxyphenylalkylamine derivatives using the mouse HTR paradigm. Although these compounds interact with the 5-HT_2A_ receptor in vitro (Fantegrossi et al. 2005; Luethi et al. 2018), not all 5-HT_2A_ agonists act as psychedelic drugs in humans. Most of the compounds induced head twitches in mice, indicating they have an LSD-like behavioral profile. Although 2C-T had relatively low potency in mice, adding an α-methyl group increased potency fivefold. Potency was also increased when the 4-methylthio-group was lengthened by one to three methylene units. Fluorination of the 4-position alkyl chain, however, was detrimental for activity, as was replacement of a saturated *n*-propylthio group with an unsaturated allylthio substituent. In addition, compounds containing a 4-benzylthio group showed little or no effect in the HTR paradigm, which is consistent with evidence that relatively bulky 4-substituents can dampen agonist efficacy at the 5-HT_2A_ receptor (Dowd et al. 2000; Luethi et al. 2018).

Although we did not identify the receptor responsible for the effect of 2C-T and its analogs in the HTR experiments, the responses observed in Fig. 2 are likely mediated by 5-HT_2A_ receptor activation. There is considerable evidence that the HTR induced by psychedelic drugs occurs as a consequence of agonist effects on 5-HT_2A_ (Gonzalez-Maeso et al. 2007; Halberstadt and Geyer 2014; Halberstadt et al. 2011). For example, the ability of 2C-T-7 to induce the HTR is blocked in mice pretreated with the highly selective 5-HT_2A_ antagonist M100907 (Fantegrossi et al. 2005). Consistent with the anticipated receptor mechanism for the HTR induced by 2C-T and its analogs, all of the molecules in Fig. 2 bind to 5-HT_2A_ with nanomolar affinity and act as agonists or partial agonists in calcium flux assays.

The pattern of activity of 2C-T and its analogs in the HTR experiments is roughly consistent with their structure–activity relationships at the 5-HT_2A_ receptor (see Fig. 1 for an overview). First, simple homologation of the 4-position substituent in 2C-T has parallel effects on HTR potency and 5-HT_2A_ binding affinity. 2C-T induces the HTR with lower potency (ED_50_ = 13.8 µmol/kg) than its 4-ethylthio (ED_50_ = 1.44 µmol/kg), 4-*n*-propylthio (ED_50_ = 2.12 µmol/kg), and 4-*n*-butylthio (ED_50_ = 3.27 µmol/kg) homologues. Likewise, in radioligand binding assays, 2C-T reportedly displaces [^3^H]ketanserin binding to the human 5-HT_2A_ receptor with *K*_i_ = 49 nM, whereas 2C-T-2 (*K*_i_ = 9 nM; Rickli et al. 2015), 2C-T-7 (*K*_i_ = 5.3 nM) and 2C-T-19 (*K*_i_ = 6.9 nM) have higher affinity (Luethi et al. 2018). Second, fluorination of the 4-position substituent in 2C-T analogs also has a paralleling influence on trends for HTR potency and 5-HT_2A_ binding affinity. Mono- or difluorination reduces potency in the HTR paradigm: 2C-T-2 (ED_50_ = 1.44 µmol/kg) has higher potency compared to its monofluorinated analog 2C-T-21 (ED_50_ = 5.58 µmol/kg) and its difluorinated analog 2C-T-21.5 (ED_50_ = 9.97 µmol/kg). Similarly, 2C-T-7 (ED_50_ = 2.12 µmol/kg) has higher potency than its monofluorinated homologue 2C-T-28 (ED_50_ = 3.49 µmol/kg), although the effect is less dramatic. Fluorine substitution also has detrimental effects on 5-HT_2A_ binding: 2C-T-2 has higher affinity (*K*_i_ = 9 nM; Rickli et al. 2015) than either 2C-T-21 (*K*_i_ = 27 nM; present results) or 2C-T-21.5 (*K*_i_ = 14 nM; Luethi et al. 2018), and 2C-T-7 (*K*_i_ = 5.3 nM; Luethi et al. 2018) has higher affinity than 2C-T-28 (*K*_i_ = 17 nM; Luethi et al. 2018). Third, replacement of the 4-propylthio group in 2C-T-7 with a 4-allylthio group in 2C-T-16 reduced HTR potency and 5-HT_2A_ affinity: 2C-T-16 has about 50% of the potency of 2C-T-7 in the HTR paradigm (Table 2) and 2C-T-16 binds to the 5-HT_2A_ receptor with *K*_i_ = 9.2 nM whereas as shown above 2C-T-7 has approximately twofold higher affinity (Luethi et al. 2018).

For 2,4,5-trisubstituted phenylalkylamines with a 2,5-dimethoxy substitution pattern, the identity of the 4-position substituent has a profound influence on activity at the 5-HT_2A_ receptor. Phenylalkylamines containing a lipophilic group in the 4-position typically have enhanced affinity, whereas molecules with polar substituents have low affinity (Glennon et al. 1992b; Nelson et al. 1999; Nichols et al. 1994; Seggel et al. 1990). In compounds containing a linear lipophilic chain at C4 of the aromatic nucleus, such as an *n*-alkyl group, 5-HT_2A_ affinity tends to increase in proportion to chain length. There are, however, steric constraints on activity; 5-HT_2A_ agonist efficacy starts to drop off if the C4 substituent is greater than 3–6 atoms long. Bulky or branched groups, such as *sec*-butyl or *tert*-butyl, are also detrimental for agonist activity (Glennon et al. 1982; Oberlender et al. 1984). In simulated docking studies, when 2,4,5-trisubstituted phenylalkylamines and their *N*-benzyl (NBOMe) analogs bind to the 5-HT_2A_ receptor, the protonated amine interacts with Asp155^(3.32)^, there is a π-π interaction between the aromatic ring and Phe340^(6.52)^, and the 2- and 5-methoxy groups interact with Ser159^(3.36)^ and Ser239^(5.43)^ via hydrogen bonds (Braden and Nichols 2007; Braden et al. 2006; Chambers and Nichols 2002; Isberg et al. 2011). These predictions are supported by site-directed mutagenesis (Braden and Nichols 2007; Braden et al. 2006; Choudhary et al. 1993; Wang et al. 1993). Based on the relationship between 4-substituent lipophilicity/steric bulk and activity at 5-HT_2A_, there has been considerable speculation that the substituent is accommodated by a specific hydrophobic region of the receptor (Glennon and Seggel 1989; Kier and Glennon 1978; Nichols et al. 1977); the exact location of the hydrophobic site, however, could not be determined based on simulated docking. Recently, the crystal structure of 25CN-NBOH bound to a complex between h5-HT_2A_ receptor and an engineered Gαq heterotrimer complex was solved using cryo-EM (Kim et al. 2020). When 25CN-NBOH binds to the 5-HT_2A_ receptor, the 4-position cyano group occupies a hydrophobic pocket formed between V235, G238, and S239 in TM5 (Kim et al. 2020). The 4-sulfur substituents in 2C-T and analogs may interact with the same hydrophobic region of the 5-HT_2A_ receptor as the 4-CN in 25CN-NBOH; in that case, the identity of the C4 substituent could potentially affect activity at 5-HT_2A_ through multiple mechanisms. Nonpolar interactions between lipophilic substituents and solvated or unsolvated hydrophobic pockets on protein surfaces are often energetically favorable (Bissantz et al. 2010; Chandler 2005). Van der Waals interactions between the 4-substituent and residues lining the hydrophobic pocket could also contribute binding energy. In addition, the 4-position substituent could influence or perturb the ability of other parts of the molecule to interact with the receptor. For example, the 4-substituent could alter the electron density of the aromatic ring, potentially affecting the strength of the interaction with Phe340. Likewise, Nichols (2012) has speculated that the 4-substituent may influence the overall position of 2,4,5-trisubstituted phenylalkylamines in the binding pocket by acting as a spacer, potentially impacting a range of interactions required for ligand recognition and receptor activation.

While fluorination reduces the potency of 2C-T-2 and 2C-T-7 in the HTR paradigm, the same is not true for activity in humans. 2C-T-2 is reportedly active as a psychedelic drug at p.o. doses ranging from 12 to 25 mg (Shulgin and Shulgin 1991); similarly, the monofluorinated analog 2C-T-21 is active at 8–20 mg and the difluorinated analog 2C-T-21.5 is active at 12–30 mg. Likewise, 2C-T-7 and its monofluorinated derivative 2C-T-28 are active at p.o. doses of 10–30 mg and 8–20 mg, respectively (Shulgin and Shulgin 1991; Trachsel et al. 2013). Fluorination therefore does not appear to alter the potency of 2C-T-2 and 2C-T-7 as psychedelic drugs. There is normally a close correlation between the effects of psychedelic drugs in the mouse HTR paradigm and their activity in humans (Halberstadt et al. 2020); it is not clear why there is a discrepancy with the fluorinated derivatives of 2C-T-2 and 2C-T-7. Species differences at the receptor level are probably not involved because fluorination also reduces the affinity of 2C-T-7 for the human 5-HT_2A_ receptor (Luethi et al. 2018), which is consistent with the effect of fluorination on potency in the HTR assay. The most likely explanation for the potency difference, therefore, is that fluorination affects the distribution or clearance of 2C-T-2 and 2C-T-7 in a species-specific manner. 2C-T-2 and 2C-T-7 are metabolized by multiple routes, including sulfoxidation, *S*-dealkylation, *N*-acetylation, *β*-hydroxylation, and oxidative deamination (Kanamori et al. 2007, 2016; Theobald et al. 2005a, 2005b). Fluorination could alter the enzymatic clearance of 2C-T-2 and 2C-T-7 to different degrees in mice and humans, potentially causing the in vivo potencies of the fluorinated derivatives to diverge in those species. Alternatively, the in vivo potency differences could reflect species differences in CNS bioavailability. Some drugs are actively transported into the brain by a proton antiporter (Andre et al. 2009; Chapy et al. 2015; Cisternino et al. 2013). To our knowledge, it is unknown whether 2C-T-2 and 2C-T-7 undergo active transport into the brain, but if such a process does occur then fluorination could potentially alter their affinity for the transporter (and therefore their CNS delivery) in a species-specific manner.

Compounds containing a 4-alkylthio substituent induced robust behavioral responses, whereas the presence of a 4-benzylthio group was detrimental for activity. 2C-T-33 did not produce a significant increase in HTR counts compared to vehicle-treated mice. 2C-T-27 was active, but the magnitude of the response was relatively weak. 2C-T-27 and 2C-T-33 have high affinity for the 5-HT_2A_ receptor (*K*_i_ = 1.6 nM and 1.7 nM, respectively) but act as low efficacy partial agonists in 5-HT_2A_ calcium flux assays (Luethi et al. 2018). In HTR studies performed with a series of 5-HT_2_ agonists in rats, the magnitude of the behavioral response (the maximum number of head-twitch counts) induced by each compound was correlated with their efficacy at the 5-HT_2A_ receptor (Vickers et al. 2001). If the same relationship extends to 2C-T derivatives then 2C-T-27 and 2C-T-33 may not activate 5-HT_2A_ to the extent necessary to induce a robust behavioral response in HTR experiments. Similar to 2C-T-27 and 2C-T-33, other 2,4,5-trisubstituted phenylalkylamines containing large 4-substituents such as phenylpropyl, benzyl, phenyl, hexyl, or octyl retain affinity for the 5-HT_2A_ receptor but act as antagonists or weak partial agonists (Dowd et al. 2000; Kolaczynska et al. 2019; Luethi et al. 2019; Nelson et al. 1999; Seggel et al. 1990). As noted above, in the published cryo-EM crystal structure of 25CN-NBOH bound to h5-HT_2A_, the 4-cyano group occupies a hydrophobic pocket between ECL2 and TM5 (Kim et al. 2020). The volume of the hydrophobic cleft may not readily accommodate a benzylthio ring, so 2C-T-27 and 2C-T-33 may not adopt the same binding pose as 25CN-NBOH when they occupy the 5-HT_2A_ receptor. Along those lines, evidence has been reported that 4-(arylalkyl)-substituted phenylalkylamines defy the established structure–activity relationships for the 5-HT_2A_ receptor. While the removal of one of the methoxy groups from derivatives of 2C-H and 2,5-DMA normally reduces 5-HT_2A_ affinity to a considerable degree (Glennon et al. 1992a; Marcher-Rorsted et al. 2020), there was no loss of affinity when either the 2- or 5-methoxy group of 4-(3-phenylpropyl)-2,5-dimethoxyamphetamine was removed (Dowd et al. 2000). These SAR differences may reflect the existence of multiple binding modes for phenylalkylamines at the 5-HT_2A_ receptor.

One caveat is that the HTR experiments were only performed in male mice. For this investigation, the HTR in male C57BL/6 J mice is being used as a pharmacological assay that has considerable predictive validity for psychedelic potential and structure–activity relationships in humans and other species (Gonzalez-Maeso et al. 2007; Halberstadt et al. 2020). Although sex differences have been detected in HTR studies performed with male and female C57BL/6 J mice (Dunlap et al. 2020; Jaster et al. 2022), there is no evidence from human clinical trials that psychedelic drugs act with different potencies, produce different qualitative effects, or show pharmacokinetic differences in male and female subjects (Leary et al. 1963; Studerus et al. 2012; Dolder et al. 2016; Holze et al. 2019, 2022; McCulloch et al. 2022), so the sex differences observed in mice may not be modeling human phenomenology. Nevertheless, if humans do exhibit sex differences in the response to 2C-T derivatives then the present experiments may not accurately model those effects.

In summary, 2C-T and a large series of homologues and analogs activate the 5-HT_2A_ receptor and induce the HTR. These results are consistent with their classification as psychedelic drugs. In line with these findings, most of the compounds are known to produce psychedelic effects in humans. The activity of ALEPH, 2C-T, 2C-T-2, 2C-T-7, 2C-T-21, 2C-T-21.5, and 2C-T-28 in humans was discussed previously in this paper. In addition, 2C-T-3 (p.o. dose range = 15–40 mg), 2C-T-16 (10–25 mg), and 2C-T-27 (80–130 mg) reportedly act as psychedelic drugs in humans (Trachsel et al. 2013). Although ultimately all of these sulfur-substituted phenylalkylamines need to be studied in humans in clinical trials, these findings provide additional evidence that the compounds closely mimic the psychopharmacology of LSD and mescaline.

## Supplementary Information

Below is the link to the electronic supplementary material.

Supplementary file1 (DOCX 21 KB)
