# Supplementary material for: Use of the head-twitch response to investigate the structure–activity relationships of 4-thio-substituted 2,5-dimethoxyphenylalkylamines
Source: Psychopharmacology (Berl). 2022 Dec 7;240(1):115–26. doi: 10.1007/s00213-022-06279-2 (PMC9816194; doi:10.1007/s00213-022-06279-2)
Supplement: Supplementary file 1 — Supplementary file1 (DOCX 21 KB) [file 213_2022_6279_MOESM1_ESM.docx]

Supplementary information

Table S1. Summary of the results of the HTR experiments

| Compound | ANOVA | Dose (mg/kg) | *N* | HTR Counts (Mean ± SEM) | ED_50_ mg/kg  (95% CI) | ED_50_ µmol/kg (95% CI) |
| --- | --- | --- | --- | --- | --- | --- |
| TMA-2^a^ |  |  |  |  | 2.79 (1.94-4.01) | 12.4 (8.6-17.8) |
| ALEPH | *W*(5,10.78)=24.83, *p*<0.0001 | 0 | 5 | 4.6 ± 1.2 | 0.80 (0.53-1.20) | 2.88 (1.91-4.32) |
|  |  | 0.1 | 5 | 7.8 ± 1.2 |  |  |
|  |  | 0.3 | 5 | 19.4 ± 1.2*** |  |  |
|  |  | 1 | 5 | 72.0 ± 12.1* |  |  |
|  |  | 3 | 5 | 115.0 ± 20.9* |  |  |
|  |  | 10 | 5 | 74.0 ± 13.5* |  |  |
| 2C-T | *W*(5,11.72)=70.03, *p*<0.0001 | 0 | 7 | 5.3 ± 1.4 | 3.65 (3.09-4.32) | 13.8 (11.7-16.4) |
|  |  | 1.5 | 5 | 22.4 ± 5.9 |  |  |
|  |  | 3 | 6 | 49.3 ± 3.1**** |  |  |
|  |  | 6 | 6 | 61.0 ± 4.7*** |  |  |
|  |  | 12 | 6 | 97.2 ± 8.1*** |  |  |
|  |  | 24 | 5 | 64.8 ± 6.0** |  |  |
| 2C-T-2 | *W*(4,10.02)=18.13, *p*<0.0001 | 0 | 7 | 11.4 ± 2.3 | 0.40 (0.28-0.57) | 1.44 (1.01-2.05) |
|  |  | 0.1 | 6 | 12.2 ± 1.9 |  |  |
|  |  | 0.3 | 5 | 28.4 ± 4.7 |  |  |
|  |  | 1 | 5 | 65.2 ± 5.9** |  |  |
|  |  | 3 | 5 | 36.8 ± 11.5 |  |  |
| 2C-T-7 | *W*(4,10.12)=11.69, *p*=0.0008 | 0 | 6 | 10.8 ± 2.0 | 0.62 (0.38-0.99) | 2.12 (1.30-3.39) |
|  |  | 0.1 | 5 | 11.8 ± 1.3 |  |  |
|  |  | 0.3 | 5 | 18.4 ± 3.5 |  |  |
|  |  | 1 | 6 | 70.2 ± 10.6** |  |  |
|  |  | 3 | 5 | 58.6 ± 9.7* |  |  |
| 2C-T-3 | *W*(5,9.97)=37.16, *p*<0.0001 | 0 | 5 | 5.8 ± 1.2 | 1.86 (1.45-2.39) | 6.12 (4.77-7.87) |
|  |  | 0.3 | 4 | 11.3 ± 3.6 |  |  |
|  |  | 1 | 5 | 26.2 ± 3.4* |  |  |
|  |  | 3 | 5 | 67.6 ± 5.4** |  |  |
|  |  | 10 | 5 | 67.8 ± 7.5** |  |  |
|  |  | 30 | 5 | 2.2 ± 1.4 |  |  |
| 2C-T-16 | *W*(4,8.33)=25.78, *p*<0.0001 | 0 | 5 | 6.8 ± 1.9 | 1.68 (1.17-2.43) | 5.80 (4.04-8.38) |
|  |  | 1.5 | 5 | 49.6 ± 11.1 |  |  |
|  |  | 3 | 5 | 101.6 ± 20.4* |  |  |
|  |  | 6 | 5 | 100.6 ± 9.6** |  |  |
|  |  | 12 | 5 | 51.2 ± 18.3 |  |  |
| 2C-T-19 | *W*(4,9.27)=4.80, *p*=0.0228 | 0 | 5 | 11.4 ± 1.5 | 1.00 (0.48-2.11) | 3.27 (1.57-6.90) |
|  |  | 0.3 | 5 | 14.2 ± 3.4 |  |  |
|  |  | 1 | 5 | 19.8 ± 1.1* |  |  |
|  |  | 3 | 5 | 26.6 ± 9.4 |  |  |
|  |  | 10 | 5 | 12.0 ± 4.1 |  |  |
| 2C-T-21 | *W*(4,10.75)=35.21, *p*<0.0001 | 0 | 6 | 5.7 ± 1.6 | 1.65 (1.19-2.27) | 5.58 (4.02-7.67) |
|  |  | 0.3 | 5 | 8.8 ± 2.1 |  |  |
|  |  | 1 | 6 | 19.8 ± 2.7** |  |  |
|  |  | 3 | 6 | 61.8 ± 5.0*** |  |  |
|  |  | 10 | 5 | 72.6 ± 9.4** |  |  |
| 2C-T-21.5 | *W*(4,9.16)=14.98, *p*=0.0005 | 0 | 5 | 9.0 ± 2.6 | 3.13 (2.48-3.93) | 9.97 (7.90-12.5) |
|  |  | 1.5 | 5 | 26.0 ± 5.4 |  |  |
|  |  | 3 | 5 | 39.4 ± 7.2* |  |  |
|  |  | 6 | 5 | 85.6 ± 9.8** |  |  |
|  |  | 12 | 5 | 40.4 ± 13.0 |  |  |
| 2C-T-27 | *W*(5,10.87)=5.05, *p*=0.0122 | 0 | 5 | 3.4 ± 0.9 | 2.48 (1.46-4.22) | 7.30 (4.30-12.4) |
|  |  | 1.5 | 5 | 6.0 ± 1.6 |  |  |
|  |  | 3 | 5 | 9.0 ± 0.8** |  |  |
|  |  | 6 | 5 | 12.8 ± 2.0* |  |  |
|  |  | 12 | 5 | 10.8 ± 3.6 |  |  |
|  |  | 24 | 5 | 5.4 ± 2.0 |  |  |
| 2C-T-28 | *W*(4,10.12)=9.73, *p*=0.0017 | 0 | 5 | 9.2 ± 2.1 | 1.08 (0.62-1.87) | 3.49 (2.00-6.04) |
|  |  | 0.3 | 5 | 7.4 ± 2.0 |  |  |
|  |  | 1 | 5 | 25.4 ± 4.1* |  |  |
|  |  | 3 | 5 | 42.0 ± 6.1* |  |  |
|  |  | 10 | 6 | 27.2 ± 7.4 |  |  |
| 2C-T-33 | *W*(4,9.35)=0.74, *p*=0.5897 | 0 | 5 | 5.6 ± 1.9 | *ND* | *ND* |
|  |  | 0.3 | 5 | 8.6 ± 2.2 |  |  |
|  |  | 1 | 5 | 5.0 ± 2.8 |  |  |
|  |  | 3 | 5 | 9.6 ± 1.7 |  |  |
|  |  | 10 | 5 | 7.8 ± 0.8 |  |  |

HTR counts were recorded over 30 min

*ND*, not determined.

**p*<0.05, ***p*<0.01, ****p*<0.001, *****p*<0.0001, significant difference vs. vehicle control (Dunnett’s T3 multiple comparisons test)
